# Hypotheses, rationale, design, and methods for prognostic evaluation of cardiac biomarker elevation after percutaneous and surgical revascularization in the absence of manifest myocardial infarction. A comparative analysis of biomarkers and cardiac magnetic resonance. The MASS-V Trial

**DOI:** 10.1186/1471-2261-12-65

**Published:** 2012-08-16

**Authors:** Whady Hueb, Bernard J Gersh, Paulo Cury Rezende, Cibele Larrosa Garzillo, Eduardo Gomes Lima, Ricardo D'Oliveira Vieira, Rosa Maria Rahmi Garcia, Desiderio Favarato, Carlos Alexandre W Segre, Alexandre Costa Pereira, Paulo Rogério Soares, Expedito Ribeiro, Pedro Lemos, Marco A Perin, Célia Cassaro Strunz, Luis AO Dallan, Fabio B Jatene, Noedir AG Stolf, Alexandre Ciappina Hueb, Ricardo Dias, Fabio A Gaiotto, Leandro Menezes Alves da Costa, Fernando Teiichi Costa Oikawa, Rodrigo Morel Vieira de Melo, Carlos Vicente Serrano, Luiz Francisco Rodrigues de Ávila, Alexandre Volney Villa, José Rodrigues Parga Filho, César Nomura, José AF Ramires, Roberto Kalil Filho

**Affiliations:** 1 From the Heart Institute of the University of São Paulo, São Paulo, Brazil; 2 Mayo Clinic, Rochester, MN, USA; 3 Av. Dr. Enéas de Carvalho Aguiar 44 AB - 114 Cerqueira César, São Paulo-SP, 05403-000, Brazil

**Keywords:** Cardiopulmonary bypass, Necrosis markers, Myocardial infarction, PCI, CABG

## Abstract

**Background:**

Although the release of cardiac biomarkers after percutaneous (PCI) or surgical revascularization (CABG) is common, its prognostic significance is not known. Questions remain about the mechanisms and degree of correlation between the release, the volume of myocardial tissue loss, and the long-term significance. Delayed-enhancement of cardiac magnetic resonance (CMR) consistently quantifies areas of irreversible myocardial injury. To investigate the quantitative relationship between irreversible injury and cardiac biomarkers, we will evaluate the extent of irreversible injury in patients undergoing PCI and CABG and relate it to postprocedural modifications in cardiac biomarkers and long-term prognosis.

**Methods/Design:**

The study will include 150 patients with multivessel coronary artery disease (CAD) with left ventricle ejection fraction (LVEF) and a formal indication for CABG; 50 patients will undergo CABG with cardiopulmonary bypass (CPB); 50 patients with the same arterial and ventricular condition indicated for myocardial revascularization will undergo CABG without CPB; and another 50 patients with CAD and preserved ventricular function will undergo PCI using stents. All patients will undergo CMR before and after surgery or PCI. We will also evaluate the release of cardiac markers of necrosis immediately before and after each procedure. Primary outcome considered is overall death in a 5-year follow-up. Secondary outcomes are levels of CK-MB isoenzyme and I-Troponin in association with presence of myocardial fibrosis and systolic left ventricle dysfunction assessed by CMR.

**Discussion:**

The MASS-V Trial aims to establish reliable values for parameters of enzyme markers of myocardial necrosis in the absence of manifest myocardial infarction after mechanical interventions. The establishments of these indices have diagnostic value and clinical prognosis and therefore require relevant and different therapeutic measures. In daily practice, the inappropriate use of these necrosis markers has led to misdiagnosis and therefore wrong treatment. The appearance of a more sensitive tool such as CMR provides an unprecedented diagnostic accuracy of myocardial damage when correlated with necrosis enzyme markers. We aim to correlate laboratory data with imaging, thereby establishing more refined data on the presence or absence of irreversible myocardial injury after the procedure, either percutaneous or surgical, and this, with or without the use of cardiopulmonary bypass.

## Background

The release of creatine phosphokinase enzymes (CPK), CK-MB isoenzyme (CK-MB), cardiac troponin, or both, in the absence of ECG waves indicating necrosis during percutaneous or surgical procedures has occurred in 10 to 35 % of cardiac patients. These indices are considered diagnostic for acute myocardial infarction (AMI) [1,2]. Such results are important, because they are similar to those found during spontaneous cardiac events in the population of patients with coronary artery disease (CAD) [3]. The release of enzyme markers of myocardial cells allows the diagnosis of AMI, when the levels of CK-MB or troponin are elevated up to 3 times above the established standard for percutaneous interventions (PCI) and up to 5 times for surgical revascularization (CABG) even in the absence of electrocardiographic changes or clinical symptoms [4,5]. On the other hand, these indices, considered indicative of AMI, have prognostic value according to the severity of the patient and the procedure performed. In this scenario, the prognosis for this event can be associated with the degree of elevation of biomarkers released and with the type of intervention applied. Also included in the prognosis of the condition are the number of arteries involved, the degree of arterial and ventricular impairment, and the type of intervention performed.

However, both invasive and noninvasive investigations during the period after the intervention have limitations related to the poor the sensitivity and specificity of the ECG and biomarkers. Although cardiac-specific biomarkers potentially have the greatest role in simple detection of myocyte necrosis, their independent utility in this setting remains inaccurate.

To increase the diagnosis, the accuracy of delayed enhancement CMR imaging (DE-CMR) can quantify reversible and irreversible myocyte necrosis.

In fact in recent studies, Pegg et al. [6] reported increased cardiomyocyte necrosis associated with the hybrid technique of on-pump beating-heart CABG both detected by DE-CMR and biomarkers. In this study, the authors had as an end point the effectiveness of biomarkers troponin I (TnI) and creatine kinase-MB isoform (CK-MB) and compared these with CMR to diagnose AMI.

Moreover, these authors evaluated the efficacy of cardiac enzymes for determination of cardiomyocyte necrosis after CABG and compared this with the efficacy of CMR [7].

In the MASS-V study, we will prospectively examine the incidence and extent of new DE-CMR-defined irreversible injury in patients undergoing PCI or CABG and correlate it with postprocedural changes in TnI and CK-MB. Our hypothesis is that the releasing of biomarkers does not necessarily leads to myocardial fibrosis or left ventricular dysfunction; and the cut-off levels to define periprocedural AMI would be different from the ones established in current guidelines.

## Methods/Design

The MASS-V Trial is an institutional project that the Heart Institute (InCor), Hospital das Clinicas, University of São Paulo designed to prospectively investigate 150 patients with multivessel coronary artery disease (CAD) with normal left ventricle ejection fraction (LVEF) and with a formal indication for CABG or PCI. Fifty patients will be operated on with cardiopulmonary bypass (CPB) and another 50 patients with the same arterial and ventricular conditions and also with a proper indication for myocardial revascularization will be operated on without the help of the CPB circuit. Another 50 patients with CAD and preserved ventricular function with a formal indication for percutaneous coronary intervention (PCI) with the use of intracoronary "stents” will be analyzed.

**Figure 1:**
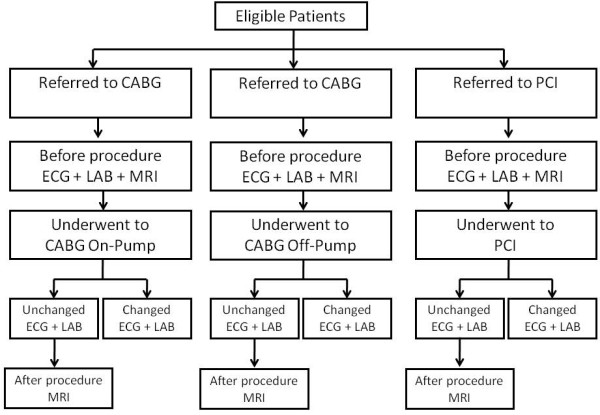
**Algorithm of MASS V Trial. **Legend: Enrollment and follow-up procedures in the arms of MASS V trial.

The inclusion criteria will determine which patients are eligible for which of the three procedures. Anginal symptoms must be stable and the LVEF preserved. The main steps of the procedures are shown in Figure 1. Exclusion criteria include the following: recent myocardial infarction (≤6 months); signs of manifest or suspected infections or rheumatologic disease activity; chronic renal failure (creatinine level >2.0 mg/dL); recent (≤ 6 months) pulmonary embolism or venous thromboembolism; not signing the consent form; contraindication for the use of glycoprotein IIb/IIIa inhibitors or CMR examination, for example, a person with a pacemaker or severe claustrophobia.

All patients will undergo CMR before and after surgical or percutaneous interventions to determine the presence or absence of myocardial necrosis complications after the procedure.

In addition, we will be evaluating the release of cardiac necrosis markers immediately before and after each procedure. The measurement of necrosis markers in the series will be conducted 6, 12, 24, and 36 hours after the percutaneous intervention. For the surgical procedures, dosage series will expand from 48 to 72 hours.

### CMR protocol

The patients will be studied in a 1.5-T clinical MR scanner (Philips Achieva), and steady-state free-procession cine images will be acquired in 2 long-axis (2 and 4 chambers view) and 8 to 10 short-axis views of the left ventricle. A gadolinium-based contrast agent (Gadoterate meglumine Gd-DOTA, Guerbet SA, France) will then be injected intravenously (0.1 mmol per kilogram of body weight), and contrast-enhanced images will be acquired after a 5- to 10-minute delay with the use of an inversion-recovery segmented sequence. Contrast-enhanced images will be acquired in long- and short-axis planes identical to the cine images. Typical voxel size will be 1.6x2.1x8mm, with a reconstruction matrix of 528 and a reconstructed voxel size of 0.6 mm. The method for acquiring and analyzing CMR is standardized in our service and is reproduced according to conventional techniques [2,8].

### Delayed enhancement

Delayed enhancement of cardiac magnetic resonance will be performed with a phase-sensitive inversion recovery (PSIR) sequence (repetition time 6.1, echo time 3.0 ms, voxel size 1.6x2.1x8mm, flip angle 25^o^) following a 5-min time delay after the administration of 0.1 mmol/kg contrast agent (Gadoterate meglumine Gd-DOTA, Guerbet SA, France). Images will be acquired in two long-axis planes and in a short-axis stack covering the entire left ventricle. The inversion time will be meticulously adjusted throughout the acquisition to obtain optimal nulling of remote normal myocardium. The slice thickness at the apex will be reduced to 5 mm to avoid a partial volume effect [7].

### CMR postprocessing and data analysis

All areas of late gadolinium-DTPA hyperenhancement will be quantified with a computer-assisted planimetry program - CMR42 v. (Circle Cardiovascular Imaging, Calgary, Canada) and interpreted by two experienced observers blinded to the interventional technique and biochemical data. When measurements are different, a third observer will perform a review and a consensus will be obtained.

Hyperenhanced pixels are defined as those with image intensities >2 SDs above the mean of image intensities in a remote myocardial region in the same image. Furthermore, we prospectively identified the site of any new hyperenhancement in relation to the implanted stent. Areas of new hyperenhancement that occur in the same short-axis image as the stent will be classified as adjacent to stent injury, whereas new hyperenhancement that occurs in the myocardium distal to the stent is deemed downstream injury.

Just as with a percutaneous intervention, hyperenhanced pixels will be defined as image intensities greater than two standard deviations above the mean intensities in a remote myocardial region in the same image. Preintervention and postintervention scans will be read side by side in both surgical techniques, with and without extracorporeal circulation.

### Biochemistry

Blood samples will be collected from each patient for measurement of troponin I (TnI) and CK-MB mass immediately before PCI and 6, 12, 24, and 36 hours after the procedure. For patients undergoing coronary artery bypass surgery, on-pump or off-pump, these cardiac markers will be measured immediately before surgery and 6, 12, 24, 36, 48, and 72 hours after surgery.

The surgeon and clinical team will be blinded to the CK-MB or TnI data.

All the samples will be centrifuged at 3000 rpm for 20 minutes and analyzed within 2 hours after specimen collection. TnI and CK-MB analysis will be performed in an immunoassay analyzer (ADVIA Centaur, Siemens Health Care Diagnostics, Tarrytown, NY). According to the manufacturer, the lower limit of detection of TnI using a high-sensitivity kit, the Ultra kit, is 0.006 ng/mL, and the 99th percentile and MI reference limits are respectively 0.04 and 0.76 ng/mL. The assay precision, represented by the percentage coefficient of variation (%CV) is ≤ 10 % at 0.03 ng/mL.

The detection limit of the CK-MB mass kit is 0.18 ng/mL, and the cut off values at the 99th percentile, established by our laboratory, are 3.8 ng/mL for women and 4.4 ng/mL for men. The CVs for CK-MB mass, as specified by the manufacturer, are 3.91 % at 3.55 ng/mL and 3.61 % at 80.16 ng/Ml.

### Statistical analysis

Values will be expressed as mean (±SD) or median (interquartile range) as appropriate. The paired-sample *t* test and the unpaired-sample *t* test will be used to compare means within the study group or between subgroups. *χ*^2^ statistics with Fisher’s exact test will be used for comparison of discrete variables. Continuous variables that were not distributed normally will be compared with the Mann–Whitney *U* test, and correlation between such variables will be made with the Spearman rank test. Binary logistic regression will be performed to determine which clinical and angiographic parameters predict the likelihood of myocardial hyperenhancement. Multivariate logistical regression will be used to assess the relative contribution of various clinical and angiographic variables to the presence of new hyperenhancement after PCI or CABG. A probability value of <0.05 is considered statistically significant.

### Trial outcomes

Primary outcome considered is overall death in a 5-year follow-up. Secondary outcomes are levels of CK-MB isoenzyme and I-Troponin in association with presence of myocardial fibrosis and left ventricle ejection dysfunction assessed by CMR.

## Discussion

The release of biomarkers of myocardial necrosis occurs in most patients who undergo mechanical interventions of the heart, both CABG and PCI. Among the causes of this release are the trauma caused by the handling organ, contact of blood with the bypass circuit, aortic cross clamping, and reperfusion injury. On the other hand, periprocedural myocardial injury during PCI can result from procedural complications, such as distal embolization, side-branch occlusion, coronary dissection, and disruption of collateral flow.

The clinical significance and long-term prognosis of this condition continues to be debated. Initial short-term follow-up found no increase in the incidence of cardiac events; however, further large prospective trials have suggested that elevation of biomarkers of myocardial necrosis after PCI or CABG is clinically relevant. In addition, outcomes after CABG or PCI with very high procedural TnI or CK-MB levels have prognostic implications considered similar to those of spontaneous acute myocardial infarction.

Delayed enhancement CMR imaging can quantify irreversible myocyte necrosis for the identification of subedocardial infarction.

However, uncertainties remain about the mechanisms and functional significance of the release of cardiac biomarkers and the direct relationship between procedure-related troponin and CK-MB increase and the degree of myocardial tissue loss.

### Ethical considerations

MASS-V trial will be conducted in accordance with the principles of the Declaration of Helsinki and with laws and regulations of our country. The Ethics Committee of the Heart Institute of the University of São Paulo, Brazil, approved the study protocol. The attending physician will obtain written informed consent from the all study participants.

### Trial status

This is a proposed trial and the enrollment process has not started until the date of manuscript submission.

## Abbreviations

AMI: Acute Myocardial Infarction; CABG: Coronary Artery Bypass Graft; CMR: Cardiac Magnetic Resonance; CPB: Cardiopulmonary Bypass; CPK: Creatine Phosphokinase Enzymes; CK- MB: Creatine Kinase Isoenzyme; CAD: Coronary Artery Disease; CPB: Cardiopulmonary Bypass; CV: Coefficient of Variation; DE-CMR: Delayed Enhancement CMR imaging; LVEF: Left Ventricle Ejection Fraction; MASS: Medicine, Angioplasty, or Surgery Study; PCI: Percutaneous Coronary Intervention; SD: Standard Deviation; TnI: Troponin I.

## Competing interests

None of the authors of the MASS-V Trial has a financial or any other relation that would pose a conflict of interest.

## Authors’ contributions

Each of the authors has made substantial contributions to this manuscript, either in conception and design or in the drafting of the article and critical revision for important intellectual content. Specifically, study concept and design: WH, BJG, CLG, ACP, PCR, CN, CCS, JAFR, RKF. Acquisition of data: FTCO, RMVM, LMAC, ER, PL, NS, FJ, ACH, CN, CCS. Analysis and interpretation of data: WH, PCR, EGL, CLG. Drafting of manuscript: Critical revision of the manuscript for important intellectual content: CN, ACP, CCS, ER, NS, FJ. Statistical expertise: ACP, DF, PL, CVSJ. Study Supervision/Support and planned Ancillaries Studies: WH, ER, PL, NGS. All authors participated in drafting and revising the manuscript, and all authors have read and approved the final manuscript.

## Author’s information

Steering Committee Members, Whady Hueb, Bernard J Gersh, Eduardo Gomes Lima, Cibele Larrosa Garzillo, Rosa Maria Rhami Garcia, Paulo Cury Rezende, Pedro Lemos, Expedito Ribeiro, Célia Cassaro Strunz, Fabio B Jatene, Noedir A G Stolf, José A F Ramires, Roberto Kalil Filho. Cardiology Committee, Whady Hueb, Eduardo Gomes Lima, Cibele Larrosa Garzillo, Paulo Cury Rezende, Fernando Teiichi Costa Oikawa, Rodrigo Morel Vieira de Melo, Leandro Menezes Alves da Costa, José A F Ramires, Roberto Kalil Filho. Hematologic Disorders Committee, Dalton A F Chamone, Roberto Abi-Rached, Alexandre Costa Pereira. Endocrinologist Committee, Rosa Maria Rhami Garcia, Raul Maranhão, Célia Cassaro Strunz. Cardiac Imaging Techniques Committee, Cesar Nomura, José Rodrigues Parga Filho, Luiz Francisco Rodrigues de Ávila, Alexandre Volney Villa. Electrocardiography and exercise stress testing Committee, Willian Chalela, Augusto H Uchida. Interventional Committee, Expedito Ribeiro, Pedro Lemos, Marco A Perin Alexandre Ciappina Hueb. Cost-effectiveness and Quality of Life Committee, Myrthes E Takiuti, Priscyla Girardi, Marcela F Silva, Ana Luiza Oliveira Carvalho, Teryo Nakano. Ancillary Studies Committee, Cesar Nomura, Alexandre Costa Pereira, Célia Cassaro Strunz, Expedito Ribeiro, Noedir A G Stolf, Fabio B Jatene. Data and Safety Monitoring Board, Eliana Lima, Laura Caringe, Marcela F Silva. Source Information, The members of the writing group, Whady Hueb, Célia Cassaro Strunz, Alexandre Costa Pereira, Expedito Ribeiro, Pedro Lemos, Cesar Nomura, Fabio B Jatene, José A F Ramires, and Roberto Kalil Filho assume all responsibility for the overall content and integrity of this article.

### Funding

The MASS-V Trial is funded in part by the Zerbini Foundation and Fundação de Amparo á Pesquisa do Estado de São Paulo (FAPESP) Number 2011/20876-2 Sao Paulo, Brazil.

## Pre-publication history

The pre-publication history for this paper can be accessed here:

http://www.biomedcentral.com/1471-2261/12/65/prepub
